# Mapping Plastic Reconstructive Surgical Needs and Access Barriers in Sub‐Saharan Africa: A Scoping Review

**DOI:** 10.1002/wjs.70484

**Published:** 2026-07-04

**Authors:** Sabina Rodriguez Velásquez, Camille Beatrice Gaza Bionda Valera, Saidu Idris Ahmad, Nante Rachel Wangi, Sara Botero Mesa, Peter Nthumba, Anne Zeidan, Pierre Quinodoz, Olivia Keiser, Lionel Dumont

**Affiliations:** ^1^ Institute of Global Health University of Geneva Geneva Switzerland; ^2^ The GRAPH Network Geneva Switzerland; ^3^ Accident and Emergency Unit Murtala Muhammad Specialist Hospital Kano Nigeria; ^4^ Department of Medicine Clinical Epidemiology Unit College of Health Sciences Makerere University Kampala Uganda; ^5^ Department of Plastic Surgery AIC Kijabe Hospital Kijabe Kenya; ^6^ Department of Plastic Surgery Vanderbilt University Medical Center Nashville Tennessee USA; ^7^ Independent Researcher; ^8^ Department of Plastic and Reconstructive Surgery La Tour Hospital Geneva Switzerland; ^9^ Department of Acute Medicine Division of Anesthesiology Hôpitaux Universitaires de Genève Geneva Switzerland

**Keywords:** barriers to surgical care, burns, congenital malformation, reconstructive surgery, sub‐saharan Africa, surgical workforce, trauma

## Abstract

**Background:**

An estimated 140 million additional surgical procedures are required annually, yet access to safe, timely, and affordable surgical care remains limited, particularly in low‐ and middle‐income countries, where the poorest 2.2 billion people receive only 3.5% of procedures. In sub‐Saharan Africa, the burden of trauma, burns, and congenital malformations far exceeds available capacity, yet evidence on plastic reconstructive surgery (PRS) remains fragmented and poorly quantified. This scoping review mapped the current state of PRS evidence, disease burden, and barriers to care in the region.

**Methods:**

We conducted comprehensive search of PubMed, Embase, and Web of Science, Bioline International, and AJOL (Oct 21, 2024) supplemented by gray‐literature screening. Studies addressing PRS‐relevant pathologies, surgical capacity, or barriers to care in sub‐Saharan Africa (excluding South Africa) were included without date or language restriction. Data were charted in Covidence and synthesized descriptively.

**Results:**

Of 11,780 records, 353 met inclusion criteria. Trauma (43.6%) and burns (40.2%) dominated reconstructive workload, with congenital anomalies reported in 33.4% of studies. Most research originated from Nigeria, Uganda, and Tanzania and was concentrated in tertiary hospitals. Workforce shortages, inadequate infrastructure, and financial barriers were the most frequent obstacles. Skin grafting and wound management comprised over one‐third of reported procedures. PRS interventions were highly cost‐effective, with estimates as low as US$33 per DALY averted.

**Conclusions:**

Reconstructive surgery in sub‐Saharan Africa remains critically under‐researched and under‐resourced. Addressing inequities requires locally adapted assessment tools, workforce expansion, and integration of PRS into national surgical plans.

## Graphical abstract

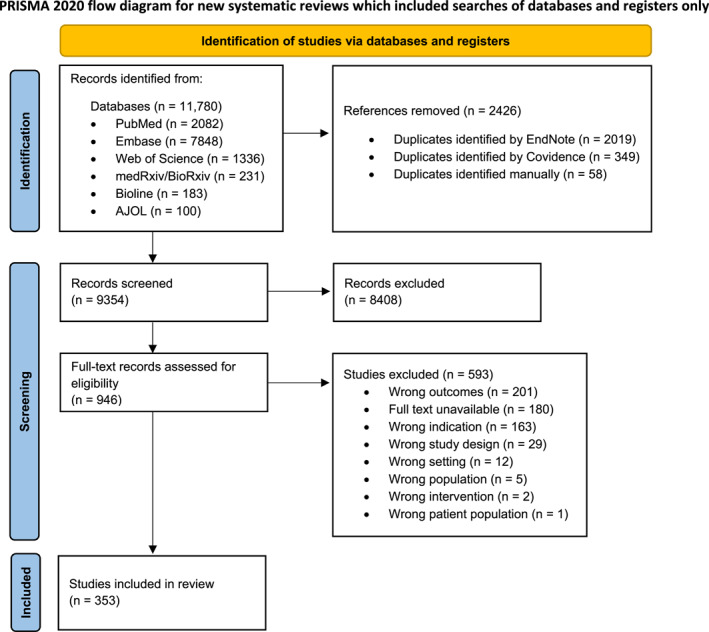

## 1 Introduction

Globally, over 140 million additional surgical procedures are required annually to meet population health needs, with demand projected to increase through 2035; however, safe, timely, and affordable surgical and anesthetic care remains limited, particularly in low‐and middle‐income countries (LMICs), where the world's poorest 2.2 billion people receive only 3.5% of all surgical procedures, highlighting substantial imbalances in surgical resource distribution [1, 2, 3, 4]. Of the global surgical disease burden, an estimated 66% is attributed to injuries, malignancy, and congenital malformations, with trauma and burns representing the greatest burden [5], many of which possibly require reconstructive intervention.

Despite the critical importance of reconstructive surgical care for managing and optimizing functional outcomes among these vulnerable populations, stark inequalities persist in surgical care distribution [6]. For instance, while North America has approximately six plastic reconstructive surgeons per million people, China and India have fewer than 1.5 per million [7], and many Sub‐Saharan African countries report fewer than one, or none at all [8], revealing dramatic disparities in specialist availability.

Disparities in surgical access are exceptionally pronounced in reconstructive surgery as the burden of disease and demand for reconstructive surgical care in post‐emergency and low‐resource settings remain poorly quantified [9]. Understanding of the sub‐Saharan African context remains limited due to insufficient data collection systems, constrained resources for reconstructive interventions, and a shortage of specialized medical personnel, resulting in a predominantly task‐shifted rather than specialist‐led workforce [3, 10]. This lack of comprehensive evidence on reconstructive surgical care presents a critical knowledge gap that hinders effective policy development and resource allocation in sub‐Saharan Africa.

No previous review has comprehensively mapped reconstructive surgical conditions, healthcare barriers, and service delivery models in Sub‐Saharan Africa.

This review aims to characterize reconstructive conditions, identify barriers to care, map evidence gaps, and inform future policy and research priorities.

## 2 Methods

The study protocol and design of this scoping review was modeled after the Arksey and O'Malley methodological framework alongside the Joanna Briggs Institute guidelines for scoping reviews [11, 12, 13]. Reporting was conducted in accordance with the Preferred Reporting Items for Systematic Reviews and Meta Analyses extension for Scoping Reviews (PRISMA‐ScR) guidelines [14].

A comprehensive protocol detailing study objectives, eligibility criteria, and methodological approach was registered on Open Science Framework on November 4, 2024 (https://osf.io/t7yb9) [15].

### 2.1 Search Strategy and Selection Criteria

Given the limited availability of plastic and reconstructive surgery (PRS)‐specific datasets, eligibility was based on conditions requiring reconstructive intervention rather than specialty designation, reflecting the task‐shifter nature of reconstructive care delivery across the region.

Eligible literature included studies involving populations in sub‐Saharan Africa with conditions potentially requiring reconstructive intervention, including injuries and trauma sustained in post‐emergency settings (e.g., armed conflict, road traffic injuries, natural disasters), burns, congenital malformations, soft‐tissue defects, and other mutilating, disabling, or deforming conditions. Studies addressing reconstructive surgical burden, healthcare capacity, hospital infrastructure, workforce characteristics, referral systems, or barriers to accessing reconstructive care were also eligible for inclusion.

Since reconstructive surgical conditions are often embedded within broader surgical, trauma, orthopedic, or emergency care literature in SSA, studies reporting conditions potentially requiring reconstructive intervention were included even when reconstructive surgery was not the primary focus of the article. From these broader studies, only reconstructive‐relevant data were extracted (e.g., cleft prevalence from congenital anomaly studies or burn contracture management from trauma literature). The inclusion of reconstructive‐relevant conditions and barriers to care was determined through consultation with expert advisors, including surgeons, anesthesiologists, and physicians on the research team.

All studies published in peer‐reviewed journals were reviewed for inclusion, except for single case studies, which were excluded due to limited generalizability. Grey literature was also eligible, including preprints, government and technical reports, theses and dissertations, policy documents, books, editorials, reviews, conference proceedings, interview transcripts, oral histories, and direct communications with authors or stakeholders.

Literature assessing reconstructive surgical techniques or best practices, as well as those solely focused on countries outside Sub‐Saharan Africa, were excluded. Of note, literature with a primary geographic and demographic focus on South Africa was excluded. In addition to South Africa's economic classification as an upper‐middle‐income country it was excluded due to its markedly different reconstructive surgical ecosystem, characterized by substantially greater specialist workforce capacity, more developed tertiary referral services, and independent specialist training pathways [16, 17, 18, 19].

Formal literature search for published articles was conducted across three electronic databases: PubMed, Embase, and Web of Science. Using key articles identified prior to literature search, a combination of database‐specific index terms such as MeSH and Emtree terms, as well as free‐text keywords related to reconstructive surgery, trauma, burns, congenital malformations, and barriers to surgical care were created. Boolean operators and truncation (e.g., reconst* surger*) were also used to enhance sensitivity.

Additional searches were conducted on Bioline International, African Journals Online (AJOL), medRxiv and bioRxiv. Additional grey literature was identified through manually screening reference lists of included studies and targeted searches of relevant organizational websites and repositories.

No publication date or language restrictions were applied. Refer to Supplementary Material 1 for the complete search strategy of each database.

Literature search was executed on 21 October 2024. Results were imported to EndNote 21 [version 21.4.0.20467] for citation management and de‐duplication.

### 2.2 Selection of Sources of Evidence

References were uploaded to Covidence for further de‐duplication as well as multi‐stage literature screening and data charting.

### 2.3 Data Charting and Analysis

Data were extracted using the Covidence Extraction 2 template with predefined study characteristics, piloted and refined by the review team. Two reviewers independently extracted data, and a third, more senior researcher, validated accuracy. Variables included study characteristics (author, date of publication, title of article, journal, peer‐review status, country of origin), study design and setting (study setting, design, population, sample size, timeframe, objectives, source of data), pathology and interventions requiring reconstructive plastic surgery (pathology, surgical procedure), epidemiological burden (Disability Adjusted Life Years, prevalence, incidence), healthcare capacity (surveillance and health information systems, provider demographics, hospital infrastructure, hospital coverage area), and barriers to care. A final summary of all included studies is provided in Supplementary Material 2.

### 2.4 Synthesis of Results

Given the broad scope and heterogeneity of the literature, a meta‐analysis, cost‐effectiveness, and precise burden estimation were not performed. Instead, descriptive statistics such as raw counts and percentages were utilized to summarize study characteristics, reported burden estimates of reconstructive surgical pathologies, and types of reconstructive surgery performed. Thematic coding was applied to qualitative data to identify patterns in reconstructive surgical capacity, healthcare provider characteristics, and barriers to reconstructive surgical care. All analyses and visualizations were conducted using R (version 2025.05.1 + 513).

## 3 Results

### 3.1 Selection of Sources of Evidence

**FIGURE 1:**
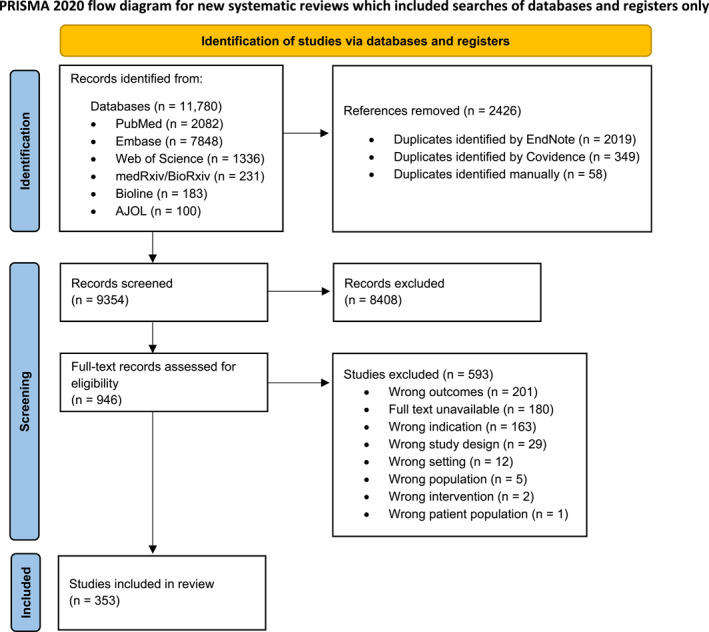
PRISMA 2020 flow diagram showing the selection process for studies included in the scoping review.

Database search retrieved a total of 11,780 records, which was reduced to 9354 records after de‐duplication. Of these, 353 studies met the eligibility criteria and were included for assessment (see Figure 1). Most studies were published in English, though French‐language publications were also included.

### 3.2 Study Characteristics

Among 353 studies assessed, most utilized cross‐sectional study designs (43.6%), followed by cohort studies (15.9%), and case series (12.8%). Descriptive studies accounted for 7.9%, literature reviews for 6.5%, and economic evaluations for 3.7%. Additional designs included prospective studies (3.4%), text and opinion pieces (2.3%), mixed‐methods studies (1.7%), qualitative research (1.1%), case‐control studies (0.8%), and other designs (0.3%). Over half of the studies (59.3%) were conducted in tertiary or referral hospitals, with smaller proportions in non‐healthcare settings (12.2%), NGO or humanitarian hospitals (6.7%), and primary care facilities (1.8%). Most relied on secondary data (53.6%), followed by primary (29.2%) and mixed sources (13.2%). More than half of first authors were affiliated with African institutions, while about one‐third were from Western institutions, as seen in Table 1.

**TABLE 1:** Study characteristics (*N* = 353 studies).

Study characteristics (*N* = 353 studies)		
Category	Variable	*n* (%)
Study design	Cross‐sectional study	154 (43.6)
	Cohort study	56 (15.9)
	Case series	45 (12.7)
	Descriptive study	28 (7.9)
	Literature review	23 (6.5)
	Economic evaluation	13 (3.7)
	Prospective study	12 (3.4)
	Text and opinion	8 (2.3)
	Mixed‐methods study	6 (1.7)
	Qualitative research	4 (1.1)
	Case‐control study	3 (0.8)
	Other	1 (0.3)
Study setting^a^	Tertiary/referral hospital	195 (59.3)
	Non‐healthcare facility	42 (12.8)
	Secondary hospital	30 (9.1)
	NGO/faith/humanitarian hospital	27 (8.2)
	Mixed facility levels	15 (4.6)
	Unspecified facilities	9 (2.7)
	Primary care facility	7 (2.1)
	Field/military hospital	4 (1.2)
Source of data^b^	Secondary data	187 (53.6)
	Primary data	102 (29.2)
	Mixed sources	46 (13.2)
	Unclear	14 (4.0)
Patients (age groups)^c^	All ages	207 (68.5)
	Pediatric	79 (26.2)
	Adult	10 (3.3)
	Unspecified	6 (2.0)
Author affiliation region	African country	189 (53.5)
	Western country	129 (36.5)
	African + Western country	30 (8.5)
	Asian country	5 (1.4)

^a^ *N* = 329 studies.

^b^ *N* = 349 studies.

^c^ *N* = 302 studies.

**FIGURE 2:**
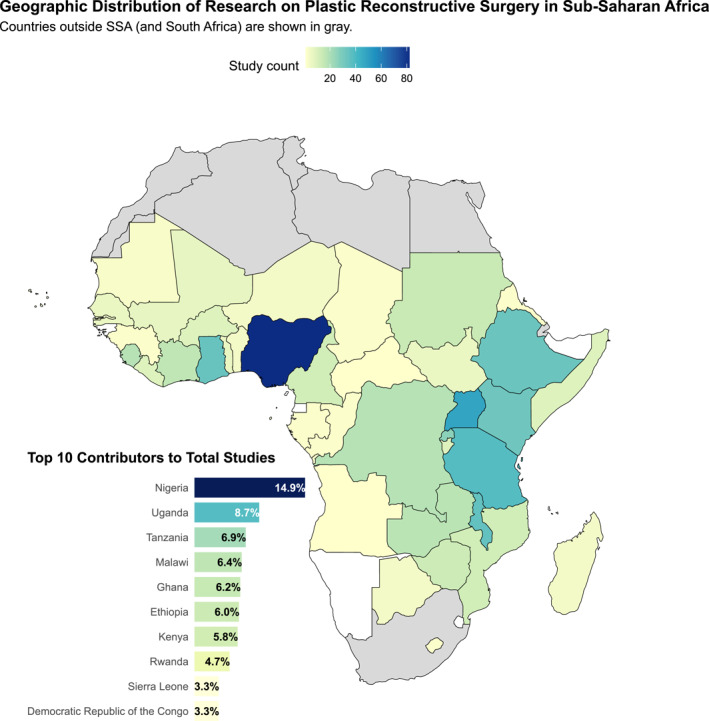
Choropleth map of geographical distribution of included studies per country.

Geographic distribution of research output reveals varied activity across the Sub‐Saharan African region. As shown in Figure 2, Nigeria contributed the largest proportion of literature, accounting for 14.9% of all studies assessed, followed by Uganda with 8.7% and Tanzania with 6.9%. The overall study distribution indicates a concentration of research in West and East African countries, with relatively fewer studies conducted in other regions.

### 3.3 Reconstructive Surgical Pathologies

Out of the 353 studies included in this scoping review, 521 pathology counts were extracted. A detailed breakdown of the specific pathologies encompassed within each general category is presented in Table 2. Trauma was the most frequently reported category (43.6%) with burn‐related pathologies being the second most frequently reported category (40.2%). Congenital malformations were reported in 118 studies (33.4%), predominantly focusing on cleft lips and palate (75.7%). Infectious (15.3%) and neoplastic conditions (11.1%) were also reported.

**TABLE 2:** Pathologies requiring plastic reconstructive surgery.

Pathologies requiring plastic reconstructive surgery				
Category	Pathology	*N*	% Within domain	% overall
Trauma	Severe injuries	42	27.45	9.50
	General fractures	36	23.53	8.14
	Soft tissue injuries	36	23.53	8.14
	Scars	13	8.50	2.94
	Open fractures	10	6.54	2.26
	Road traffic injuries	9	5.88	2.04
	Ulcers	4	2.61	0.90
	Polytrauma	3	1.96	0.68
Burns	Acute burns	99	86.09	22.40
	Post‐burn contractures and scars	16	13.91	3.62
Congenital malformations	Cleft lip and palate	78	75.73	17.65
	Clubfoot	14	13.59	3.17
	Polydactyly & syndactyly	11	10.68	2.49
Infectious conditions	Noma	16	53.33	3.62
	Buruli ulcer	8	26.67	1.81
	Necrotizing fasciitis	6	20.00	1.36
Neoplastic conditions	Cancer	11	100.00	2.49
Other	Other trauma	15	50.00	3.39
	Other infectious conditions	5	16.67	1.13
	Other neoplastic conditions	4	13.33	0.90
	Other congenital malformations	3	10.00	0.68
	Other	2	6.67	0.45
	Other burns	1	3.33	0.23

*Note:* Counts and percentage of specific pathologies and category (% within the specific domain, and % overall included literature).

The studies reviewed reported diverse epidemiological data on reconstructive plastic surgery in Sub‐Saharan Africa, covering both treated and untreated conditions across age groups. Despite heterogeneity in country setting, facility type, and methodologies, trauma, burns, and other acquired soft‐tissue injuries consistently represented the dominant reconstructive workload in the region. In Nigeria, 87.5% of emergency admissions were trauma‐related, while elective operations were largely composed of congenital deformity repair (31.6%), tumor excision (18.7%), burn reconstruction (17.3%), and esthetic procedures (12.2%) [20]. Parallel findings were observed elsewhere. In Mozambique, burns comprised 44% of 455 cases, with neoplasms (17%) and congenital anomalies (1%) following [21]. Collectively, these findings highlight that PRS services in the region are primarily oriented toward acute, acquired conditions, especially trauma and burns.

### 3.4 Surgical Procedures

A total of 568 categorized surgical procedures were identified in 244 (69%) studies. The most common were skin grafting (22.9%) and wound management (17.1%), followed by amputation or limb salvage (11.8%) and cleft and craniofacial surgery (11.6%).

### 3.5 Burden of Reconstructive Conditions

Some studies included in this review reported disability‐adjusted life year (DALY) estimates for several reconstructive surgical conditions in sub‐Saharan Africa. Their impact, measured in disability‐adjusted life years (DALYs), was estimated by one study to contribute to 38 DALYs per 1000 population in Africa [22]. Available evidence suggested that burn injuries represented one of the largest sources of conditions requiring reconstructive surgery, with estimated burdens ranging from 195 to 257 DALYs per 100,000 population in Rwanda, Sierra Leone, and Uganda [23]. Congenital anomalies, particularly cleft lips and palate, was reported to account for 13.1 DALYs per 100,000 population in sub‐Saharan Africa, with the highest national burdens in Somalia (33.3), Niger (28.3), Chad (23.2), Burkina Faso (23.1), and Mali (22.4), in another of the included studies [24]. Beyond congenital and burn‐related conditions, other pathologies, such as traumatic injuries and Noma, were reported to contribute significantly to surgical DALYs in the region [25, 26, 27].

### 3.6 Cost Effectiveness of Reconstructive Surgery

Studies from sub‐Saharan Africa show that reconstructive surgery is highly cost‐effective, averting significant disability at low cost. In Sierra Leone, procedures for burns, wounds, and contractures cost $32.78 per DALY, among the most cost‐effective interventions in the region [28].

In refugee settings, plastic surgery ranked among the most efficient specialties, with costs of $40–88 per DALY [29]. Cleft repair programs, including Smile Train, have reported similar impact at $72–134 per DALY, averaging 1.2–10.7 DALYs averted per procedure in sub‐Saharan Africa [30, 31, 32].

### 3.7 Surgical Capacity

A total of 186 studies reported on surgical capacity. Out of these, seven studies used standardized assessment tools, including the WHO Situational Analysis and PediPIPES Index, to identify personnel and infrastructure gaps [33, 34, 35, 36, 37, 38, 39].

Studies using standardized assessment tools revealed widespread workforce shortages. In Malawi, Zambia, and Tanzania, hospitals lacked specialist surgeons and physician anesthesiologists, relying instead on medical officers and nurse anesthetists [37]. Similar deficits were reported in Uganda, Cameroon, and Somalia, with fewer than one surgeon or anesthesiologist per 100,000 people [33].

Hospital capacity was limited, with few operating rooms and frequent shortages of oxygen, anesthesia equipment, and running water [33, 34, 35, 37, 39]. ICUs, emergency departments, or post‐anesthesia care units were uncommon [37].

Despite these constraints, many district hospitals provided essential care such as suturing, abscess drainage, circumcision, wound debridement, and acute burn management [35]. More complex procedures like skin grafts, contracture release, and fracture repair were largely confined to tertiary centers [37].

### 3.8 Barriers to Care

Barriers to reconstructive surgery were reported across multiple domains. Table 3 provides a breakdown of reported barriers. Health system challenges were most frequent (33.9%), particularly infrastructure gaps and human resource shortages. Studies described limited operating time, inadequate sterilization, and shortage of equipment such as dermatomes and skin meshers, which hinder burn and trauma care.

**TABLE 3:** Barriers to plastic and reconstructive surgical care.

Domain	Barrier	n	% Within domain	% Overall
Health system	Infrastructure limitations	144	51.8	17.5
	Human resource shortages	78	28.1	9.5
	Health system inefficiencies	35	12.6	4.3
	Referral system issues	16	5.7	1.9
	Other	5	1.8	0.6
	Sub‐total	278	100.0	33.9
Financial	Direct/Indirect medical costs	125	89.9	15.2
	Insurance‐related barriers	14	10.1	1.7
	Sub‐total	139	100.0	16.9
Patient‐related	Perceived need for surgery	55	44.4	6.7
	Health literacy	39	31.4	4.8
	Fear of surgery or anesthesia	18	14.5	2.2
	Comorbidities	11	8.9	1.3
	Other	1	0.8	0.1
	Sub‐total	124	100.0	15.1
Geographic/Accessibility	Physical distance	47	47.0	5.7
	Transportation issues	32	32.0	3.9
	Rural versus urban disparities	20	20.0	2.4
	Other	1	1.0	0.1
	Sub‐total	100	100.0	12.2
Educational/Training	Continuing medical education	67	91.8	8.2
	Surgeon training gaps	3	4.1	0.4
	Other	3	4.1	0.4
	Sub‐total	73	100.0	8.9
Sociocultural	Stigma and cultural beliefs	40	72.7	4.9
	Lack of social support	13	23.7	1.6
	Sex‐related barriers	1	1.8	0.1
	Other	1	1.8	0.1
	Sub‐total	55	100.0	6.7
Policy/Governance	Regulatory issues	26	66.7	3.2
	Funding gaps	13	33.3	1.6
	Sub‐total	39	100.0	4.8
Other	Political instability	7	53.8	0.9
	Other	6	46.2	0.7
	Sub‐total	13	100.0	1.6

*Note:* Counts and percentage of mentions by barrier domain and category (% within the specific domain, and % overall included literature).

Financial barriers followed (16.9%), driven by direct medical costs and systemic underfunding, delaying procedures like skin grafting [40]. Patient‐related barriers (15.1%) included low perceived need and poor health literacy, while geographic barriers (12.2%) reflected distance and transportation issues. Less common obstacles included training deficits (8.9%), sociocultural factors (6.7%), and governance‐related obstacles (4.8%).

#### 3.8.1 Availability

Availability barriers were the most frequently reported and centered on health system limitations. Studies described unreliable electricity and water, shortage of essential supplies, and inadequate access to PRS‐specific tools such as dermatomes and Humby knives. Surgeons also reported insufficient plastic surgery coverage, especially for procedures requiring flap and soft‐tissue reconstruction, citing lack of senior support and training opportunities. Although general surgical capacity has improved in some settings, reconstructive procedures like skin grafting and fracture fixation remained scarce, especially in district hospitals [41]. Persistent shortages of blood, diagnostics, and trained staff prevent comprehensive burn and trauma care, while PRS cases are often managed by orthopedic or general surgeons due to lack of specialists. This lack of expertise, coupled with inadequate infrastructure, restricted safe performance of complex reconstructions [42, 43].

Inefficient referral systems between care levels further aggravated delays in accessing appropriate surgical care [44, 45, 46, 47, 48, 49].

#### 3.8.2 Accessibility

Most people in sub‐Saharan Africa live in rural areas, while surgical services are concentrated in cities. Patients often face long travel distances, and inadequate transportation networks, preventing patients from reaching hospitals within the recommended two‐hour window for emergency surgical care. Several articles also highlighted that many patients were unaware that surgical treatments exist for their conditions, with lack of patient awareness reported by 58.2% of providers surveyed regarding orofacial cleft repairs [50].

#### 3.8.3 Affordability

Financial constraints were among the most common barriers to surgical care. Direct medical expenses, including surgical fees, hospitalization, medical prescriptions, and diagnostic procedures, were major obstacles. Indirect costs, including travel, lodging, and lost income, further increased the burden, particularly for trauma and burn patients. The absence of comprehensive national insurance programs or government subsidies across various healthcare systems also meant that economic responsibility lies solely with patients and their families, discouraging many from seeking reconstructive surgery.

#### 3.8.4 Acceptability

While less frequently described, acceptability barriers strongly influenced care‐seeking behaviors. Cultural preferences for traditional healing often delayed or outright substituted formal surgical care, especially for burn patients. Mistrust of surgical procedures and limited health literacy further discouraged treatment, with many patients doubting the necessity, safety, or benefits of reconstructive interventions.

## 4 Discussion

This review demonstrates that reconstructive surgery remains an under‐researched domain in sub‐Saharan Africa. Most surgical studies and policies focus on bellwether procedures such as cesarean section, laparotomy, hernia repair, and fracture treatment [51, 52], while pathologies requiring PRS receive limited attention, contributing to the fragmented understanding of the regions' reconstructive care needs and capacity.

The paucity of data on reconstructive surgery largely reflects the absence of dedicated assessment tools. Existing instruments, such as PediPIPES and the WHO Surgical Assessment Tool, evaluate general surgical capacity but not PRS specifically. Conditions like burns or congenital malformation are often grouped under broader categories, making reconstructive data difficult to isolate. Consequently, important gaps remain in understanding the burden of PRS conditions, available infrastructure, service delivery, and workforce capacity. Furthermore, existing surgical assessment frameworks rarely address plastic and reconstructive surgery directly and may inadequately capture care processes in resource‐constrained settings, further limiting the visibility in health system planning [53, 54, 55, 56]. This limitation is compounded by severe workforce shortages, where reconstructive surgical care is frequently performed by general surgeons, orthopedic surgeons, medical officers, and non‐physician clinicians through task‐shifted models of care [57].

A notable finding emerging from this synthesis is the apparent mismatch between reconstructive surgical burden and specialist workforce availability across the region. Trauma, burns, and congenital anomalies accounted for most conditions requiring reconstructive care, yet the literature consistently described countries with fewer than one specialist reconstructive surgeon per 100,000 population and substantial reliance on task‐shifted service delivery models [58, 59]. Although this review was not designed to quantitatively assess the relationship between workforce density and disease burden at the national level, the available evidence suggests that countries facing the greatest reconstructive needs often operate with the most constrained specialist capacity. This disparity may further contribute to the limited availability of condition‐specific data and highlights the need for more robust workforce surveillance and PRS‐specific assessment frameworks.

In our review, trauma and burns were the most frequently reported PRS conditions, yet orofacial clefts dominated disease burden estimates, reflecting a narrow research focus. This pattern likely stems from the strong influence of non‐governmental organizations (NGOs) led programs, as more than 80% of organizations providing PRS outreach in low‐ and middle‐income countries mainly focused on cleft surgery through short‐term missions [60]. In West Africa, most cleft cases are still managed by temporary humanitarian missions rather than national health services, underscoring persistent gaps in sustainable PRS delivery [61]. While NGO's play an important role in expanding access to reconstructive care, the disproportionate emphasis on cleft surgery may obscure region's broader reconstructive needs and highlights the importance of integrating PRS into national surgical, obstetric, and anesthesia planning frameworks.

An imbalance was also evident in the types of PRS procedures reported. Most studies focused on skin grafting and wound care, yet these services remain inconsistently available at district hospitals. Despite widespread recognition of their benefits, many facilities treating burn patients still lack capacity to perform grafts [40, 62].

The density of research output is concentrated in a few countries, especially Nigeria, Uganda, and Tanzania, whereas large portions of Central Africa and several low‐income countries contributed little published evidence. This pattern likely reflects difference in research infrastructure rather than true variation in reconstructive surgical need and suggests that current evidence may underestimate unmet need in underrepresented settings. In addition, most of the studies included were based in urban tertiary hospitals with limited rural representation. Population‐based surveys such as SOSAS (Surgeons OverSeas Assessment of Surgical Need) have revealed that over 80% of children with surgical conditions that might require reconstruction live in rural areas [63], while PRS‐capable facilities are concentrated in major urban centers such as Lagos, Accra, Dakar, and Nairobi [8]. Among studies assessing rural district hospitals, many reported the inability to meet the high demand for trauma and reconstructive care, performing only a limited number of procedures despite substantial need [64]. In contrast to other sub‐Saharan regions, PRS availability in Central Africa remains poorly characterized due to the few studies identified in our review, although existing evidence indicates consistently low service delivery across both urban and rural settings [8]. Overall, the evidence suggests a structural inequity in reconstructive surgical access, While the greatest burden of untreated surgical conditions is often found in rural populations, specialist services, training programs, and research activities remain concentrated in urban tertiary centers. This pattern indicated that existing estimates of reconstructive need may disproportionately reflect populations already connected to healthcare systems while underrepresenting those facing the greatest barriers to care.

The available evidence shows that patients across sub‐Saharan Africa face major overlapping barriers to surgical care that extend to PRS. Limited infrastructure, workforce shortages, and inadequate anesthesia and operating capacity constrain service delivery. Because PRS requires greater specialization and resources than general surgery, weak surgical systems hinder its development. Addressing these gaps will require context‐specific assessment tools, workforce training, and integration of PRS into national surgical plans, with future research prioritizing rural populations and locally driven approaches over NGO‐led agendas.

### 4.1 Limitations

Although no formal quality appraisal was undertaken, this scoping review provides the most comprehensive synthesis to date of literature on plastic and reconstructive surgery (PRS) in sub‐Saharan Africa, drawing from multiple databases and gray literature to map the current evidence base. Nonetheless, relevant unpublished or regionally indexed studies may have been overlooked. Most included sources were small case series, single‐institution reports, or NGO publications, concentrated in a few countries and urban tertiary centers, limiting representativeness. Definitions and measurements of PRS burden and capacity varied widely, and existing assessment tools, largely adapted from high‐income settings, were not designed for PRS, reducing reliability and contextual relevance. Furthermore, the heterogeneity in reporting more detailed analyses of the relationship between disease burden and workforce capacity, while formal bibliometric analyses were beyond the scope of this review.

As a scoping review, this study was intended to map the existing evidence rather than generate pooled epidemiologic estimates or draw definitive conclusions regarding intervention effectiveness. Consequently, the findings should not be used to derive generalized estimates of disease burden, prevalence, intervention cost‐effectiveness.

Existing burden assessments, including SOSAS and hospital‐based studies, rarely distinguish reconstructive conditions, limiting accurate estimation of regional PRS need.

## 5 Conclusion

Trauma, burns, and congenital malformations are the main contributors to reconstructive surgical need in sub‐Saharan Africa, yet existing evidence remains limited to a few countries and urban centers. A persistent gap exists between the region's high PRS burden and the capacity of health systems to deliver care, particularly in rural areas facing major barriers to access and affordability. The lack of context‐specific assessment tools and reliance on Western frameworks further constrain accurate measurement and policy integration. Future work should prioritize development of context‐specific reconstructive surgery assessment frameworks to guide evidence‐based prioritization of research and investment. Strengthening PRS in the region will require locally relevant metrics, workforce expansion, decentralized services, and integration into national surgical plans to promote equitable access and sustainable improvement in reconstructive outcomes.

## Author Contributions

**Anne Zeidan:** writing – review and editing, project administration. **Saidu Idris Ahmad:** writing – original draft, data curation, writing – review and editing. **Peter Nthumba:** writing – review and editing, methodology. **Nante Rachel Wangi:** writing – original draft, writing – review and editing, data curation. **Sara Botero Mesa:** conceptualization, funding acquisition, writing – review and editing, methodology, project administration, resources. **Lionel Dumont:** supervision, conceptualization, methodology, writing – review and editing. **Pierre Quinodoz:** funding acquisition, project administration, writing – review and editing, resources. **Olivia Keiser:** conceptualization, funding acquisition, methodology, writing – review and editing, supervision. **Camille Beatrice Gaza Bionda Valera:** conceptualization, investigation, writing – original draft, methodology, validation, visualization, writing – review and editing, data curation, supervision, formal analysis. **Sabina Rodriguez Velasquez:** conceptualization, investigation, writing – original draft, methodology, validation, visualization, writing – review and editing, data curation, supervision, formal analysis.

## Funding

Representatives of the sponsor were involved in study design, data interpretation, and reviewed the manuscript before submission.

## Ethics Statement

The authors have nothing to report.

## Conflicts of Interest

The authors declare no conflicts of interest.

## Supporting information


Supporting Information S1

Supporting Information S2

## Data Availability

All data used in this study was obtained from publicly available sources. A table including all the studies included and its extracted data is available in supplemental appendices. Code for data processing used for analysis is available upon request.
