# Supplementary material for: Mapping Plastic Reconstructive Surgical Needs and Access Barriers in Sub‐Saharan Africa: A Scoping Review
Source: World J Surg. 2026 Jul 4;50(8):2348–59. doi: 10.1002/wjs.70484 (PMC13460461; doi:10.1002/wjs.70484)
Supplement: Supplementary file 1 [file WJS-50-2348-s001.docx]

**Supplementary Material 2**

Search Strategy: Building Boolean Queries

# **Database Searches**

## **PubMed**

### *(*Result numbers based on final combination search conducted on 21 October 2024)*

| Search # | Query | Results |
| --- | --- | --- |
| 1 | "Surgical Procedures, Operative"[Mesh] OR "General Surgery"[Mesh] OR "Surgery, Plastic"[Mesh] OR "Plastic Surgery Procedures"[Mesh] OR "surger*"[tiab] OR "plastic surger*"[tiab] OR "reconst* surger*"[tiab] OR "plastic reconstr* surger*"[tiab] OR "surgi* inter*"[tiab] | 4,486,040 |
| 2 | "Wounds and Injuries"[Mesh] OR "War-Related Injuries"[Mesh] OR "injury"[tiab] OR "traumatic injury"[tiab] OR "post traumatic injury"[tiab] OR ((("Wounds and Injuries"[Mesh] AND "Natural Disasters"[Mesh]) OR ("Wounds and Injuries"[Mesh] AND "War-Related Injuries"[Mesh])) OR ("Wounds and Injuries"[Mesh] AND "Accidents"[Mesh])) OR ((("Wounds and Injuries"[Mesh] AND "Accidents, Traffic"[Mesh]) OR ("Wounds and Injuries"[Mesh] AND "Accidents, Occupational"[Mesh])) OR ("Wounds and Injuries"[Mesh] AND "Accidents, Home"[Mesh])) OR "injur*"[tiab] OR "traum* injur*"[tiab] OR "post traum* injur*"[tiab] OR "postwar injur*"[tiab] OR "traffic accident* injur*"[tiab] OR "home accident*"[tiab] OR "occupation* accident*"[tiab] | 1,754,147 |
| 3 | "Burns"[MeSH] OR "burn*"[tiab] OR "burn* injur*"[tiab] OR "burn* traum*"[tiab] | 151,181 |
| 4 | "Congenital Abnormalities"[Mesh] | 674,461 |
| 5 | "mutila*"[tiab] OR "mutila* injur*"[tiab] | 8,052 |
| 6 | "disab*"[tiab] OR "disab* injur*"[tiab] OR "disab* patho*"[tiab] | 313,914 |
| 7 | "deform*"[tiab] OR "deform* injur*"[tiab] OR "deform* patho*"[tiab] | 191,342 |
| 8 | "Prevalence"[Mesh] OR "Epidemiology"[Mesh] OR "Cost of Illness"[Mesh] OR "Mortality"[Mesh] OR "Disability-Adjusted Life Years"[Mesh] OR "prevalence"[tiab] OR "burden"[tiab] OR "cost of illness"[tiab] OR "cost of care"[tiab] OR "mortality"[tiab] OR "disability adjusted life years"[tiab] OR "DALY"[tiab] OR "years of life lost"[tiab] OR "YLL"[tiab] OR "years lived with disability"[tiab] OR "YLD"[tiab] | 2,498,721 |
| 9 | "Health Services Accessibility"[Mesh] OR "health* serv* access*"[tiab] OR "Healthcare Disparities"[Mesh] OR "health* dispar*"[tiab] OR "Health Inequities"[Mesh] OR "health* ineq*"[tiab] OR "Socioeconomic Disparities in Health"[Mesh] OR "Health Disparate Minority and Vulnerable Populations"[Mesh] OR "barrier* to care*"[tiab] | 374,293 |
| 10 | "Africa South of the Sahara"[Mesh] OR "Sub-Saharan Africa"[tiab] OR "Sub Saharan Africa"[tiab] OR "Sub-Sahara Africa"[tiab] OR "Sub Sahara Africa"[tiab] OR "sub-Saharan Africa"[tiab] OR "sub Saharan Africa"[tiab] OR "ssa"[tiab] OR "SSA"[tiab] OR "Afric*"[tiab] OR "Angola"[Mesh] OR "Angola"[tiab] OR "Benin"[Mesh] OR "Benin"[tiab] OR "Dahomey"[tiab] OR "Botswana"[Mesh] OR "Botswana"[tiab] OR "Bechuanaland"[tiab] OR "Burkina Faso"[Mesh] OR "Burkina Faso"[tiab] OR "Upper Volta"[tiab] OR "Burundi"[Mesh] OR "Burundi"[tiab] OR "Urundi"[tiab] OR "Cabo Verde"[Mesh] OR "Cabo Verde"[tiab] OR "Cape Verde"[tiab] OR "Cameroon"[Mesh] OR "Cameroon"[tiab] OR "Central African Republic"[Mesh] OR "Central African Republic"[tiab] OR "Ubangi-Shari"[tiab] OR "Chad"[Mesh] OR "Chad"[tiab] OR "Comoros"[Mesh] OR "Comoros"[tiab] OR "Congo"[Mesh] OR "Congo"[tiab] OR "Republic of the Congo"[tiab] OR "Congo-Brazzaville"[tiab] OR "Cote d'Ivoire"[Mesh] OR "Cote d'Ivoire"[tiab] OR "Ivory Coast"[tiab] OR "Democratic Republic of the Congo"[Mesh] OR "Democratic Republic of the Congo"[tiab] OR "DRC"[tiab] OR "Zaire"[tiab] OR "Belgian Congo"[tiab] OR "Djibouti"[Mesh] OR "Djibouti"[tiab] OR "French Somaliland"[tiab] OR "Equatorial Guinea"[Mesh] OR "Equatorial Guinea"[tiab] OR "Spanish Guinea"[tiab] OR "Eritrea"[Mesh] OR "Eritrea"[tiab] OR "Italian Eritrea"[tiab:~0] OR "Eswatini"[Mesh] OR "Eswatini"[tiab] OR "Swaziland"[tiab] OR "Ethiopia"[Mesh] OR "Ethiopia"[tiab] OR "Abyssinia"[tiab] OR "Gabon"[Mesh] OR "Gabon"[tiab] OR "Gambia"[Mesh] OR "Gambia"[tiab] OR "Ghana"[Mesh] OR "Ghana"[tiab] OR "Gold Coast"[tiab] OR "Guinea"[Mesh] OR "Guinea"[tiab] OR "French Guinea"[tiab] OR "Guinea-Bissau"[Mesh] OR "Guinea-Bissau"[tiab] OR "Portuguese Guinea"[tiab] OR "Kenya"[Mesh] OR "Kenya"[tiab] OR "British East Africa"[tiab] OR "Lesotho"[Mesh] OR "Lesotho"[tiab] OR "Basutoland"[tiab] OR "Liberia"[Mesh] OR "Liberia"[tiab] OR "Madagascar"[Mesh] OR "Madagascar"[tiab] OR "Malawi"[Mesh] OR "Malawi"[tiab] OR "Nyasaland"[tiab] OR "Mali"[Mesh] OR "Mali"[tiab] OR "French Sudan"[tiab] OR "Mauritania"[Mesh] OR "Mauritania"[tiab] OR "Mauritius"[Mesh] OR "Mauritius"[tiab] OR "Mozambique"[Mesh] OR "Mozambique"[tiab] OR "Portuguese East Africa"[tiab] OR "Namibia"[Mesh] OR "Namibia"[tiab] OR "South West Africa"[tiab] OR "Niger"[Mesh] OR "Niger"[tiab] OR "Nigeria"[Mesh] OR "Nigeria"[tiab] OR "Rwanda"[Mesh] OR "Rwanda"[tiab] OR "Ruanda"[tiab] OR "Sao Tome and Principe"[Mesh] OR "Sao Tome and Principe"[tiab] OR "Senegal"[Mesh] OR "Senegal"[tiab] OR "Seychelles"[Mesh] OR "Seychelles"[tiab] OR "Sierra Leone"[Mesh] OR "Sierra Leone"[tiab] OR "Somalia"[Mesh] OR "Somalia"[tiab] OR "Somali Republic"[tiab] OR "Italian Somaliland"[tiab:~0] OR "British Somaliland"[tiab] OR "South Sudan"[Mesh] OR "South Sudan"[tiab] OR "Sudan"[Mesh] OR "Sudan"[tiab] OR "Anglo-Egyptian Sudan"[tiab] OR "Tanzania"[Mesh] OR "Tanzania"[tiab] OR "Tanganyika"[tiab] OR "Zanzibar"[tiab] OR "Togo"[Mesh] OR "Togo"[tiab] OR "Togoland"[tiab] OR "Uganda"[Mesh] OR "Uganda"[tiab] OR "Zambia"[Mesh] OR "Zambia"[tiab] OR "Northern Rhodesia"[tiab] OR "Zimbabwe"[Mesh] OR "Zimbabwe"[tiab] OR "Rhodesia"[tiab] OR "Southern Rhodesia"[tiab] | 684,698 |
| 11 | 1 AND 2 | 490,005 |
| 12 | 1 AND 3 | 26,053 |
| 13 | 1 AND 4 | 180,020 |
| 14 | 1 AND 5 | 3,415 |
| 15 | 1 AND 6 | 42,791 |
| 16 | 1 AND 7 | 57,101 |
| 17 | 11 OR 12 OR 13 OR 14 OR 15 OR 16 | 733,196 |
| 18 | 17 AND 8 | 74,465 |
| 19 | 17 AND 9 | 2,424 |
| 20 | 18 OR 19 | 76,168 |
| 21 | 20 AND 10 | 2,082 |

**Final PubMed Query:**

((((((((("Surgical Procedures, Operative"[Mesh] OR "General Surgery"[Mesh] OR "Surgery, Plastic"[Mesh] OR "Plastic Surgery Procedures"[Mesh] OR "surger*"[tiab] OR "plastic surger*"[tiab] OR "reconst* surger*"[tiab] OR "plastic reconstr* surger*"[tiab] OR "surgi* inter*"[tiab]) AND ("Wounds and Injuries"[Mesh] OR "War-Related Injuries"[Mesh] OR "injury"[tiab] OR "traumatic injury"[tiab] OR "post traumatic injury"[tiab] OR ((("Wounds and Injuries"[Mesh] AND "Natural Disasters"[Mesh]) OR ("Wounds and Injuries"[Mesh] AND "War-Related Injuries"[Mesh])) OR ("Wounds and Injuries"[Mesh] AND "Accidents"[Mesh])) OR ((("Wounds and Injuries"[Mesh] AND "Accidents, Traffic"[Mesh]) OR ("Wounds and Injuries"[Mesh] AND "Accidents, Occupational"[Mesh])) OR ("Wounds and Injuries"[Mesh] AND "Accidents, Home"[Mesh])) OR "injur*"[tiab] OR "traum* injur*"[tiab] OR "post traum* injur*"[tiab] OR "postwar injur*"[tiab] OR "traffic accident* injur*"[tiab] OR "home accident*"[tiab] OR "occupation* accident*"[tiab])) OR (("Surgical Procedures, Operative"[Mesh] OR "General Surgery"[Mesh] OR "Surgery, Plastic"[Mesh] OR "Plastic Surgery Procedures"[Mesh] OR "surger*"[tiab] OR "plastic surger*"[tiab] OR "reconst* surger*"[tiab] OR "plastic reconstr* surger*"[tiab] OR "surgi* inter*"[tiab]) AND ("Burns"[MeSH] OR "burn*"[tiab] OR "burn* injur*"[tiab] OR "burn* traum*"[tiab]))) OR (("Surgical Procedures, Operative"[Mesh] OR "General Surgery"[Mesh] OR "Surgery, Plastic"[Mesh] OR "Plastic Surgery Procedures"[Mesh] OR "surger*"[tiab] OR "plastic surger*"[tiab] OR "reconst* surger*"[tiab] OR "plastic reconstr* surger*"[tiab] OR "surgi* inter*"[tiab]) AND ("Congenital Abnormalities"[Mesh]))) OR (("Surgical Procedures, Operative"[Mesh] OR "General Surgery"[Mesh] OR "Surgery, Plastic"[Mesh] OR "Plastic Surgery Procedures"[Mesh] OR "surger*"[tiab] OR "plastic surger*"[tiab] OR "reconst* surger*"[tiab] OR "plastic reconstr* surger*"[tiab] OR "surgi* inter*"[tiab]) AND ("mutila*"[tiab] OR "mutila* injur*"[tiab]))) OR (("Surgical Procedures, Operative"[Mesh] OR "General Surgery"[Mesh] OR "Surgery, Plastic"[Mesh] OR "Plastic Surgery Procedures"[Mesh] OR "surger*"[tiab] OR "plastic surger*"[tiab] OR "reconst* surger*"[tiab] OR "plastic reconstr* surger*"[tiab] OR "surgi* inter*"[tiab]) AND ("disab*"[tiab] OR "disab* injur*"[tiab] OR "disab* patho*"[tiab]))) OR (("Surgical Procedures, Operative"[Mesh] OR "General Surgery"[Mesh] OR "Surgery, Plastic"[Mesh] OR "Plastic Surgery Procedures"[Mesh] OR "surger*"[tiab] OR "plastic surger*"[tiab] OR "reconst* surger*"[tiab] OR "plastic reconstr* surger*"[tiab] OR "surgi* inter*"[tiab]) AND ("deform*"[tiab] OR "deform* injur*"[tiab] OR "deform* patho*"[tiab]))) AND ("Prevalence"[Mesh] OR "Epidemiology"[Mesh] OR "Cost of Illness"[Mesh] OR "Mortality"[Mesh] OR "Disability-Adjusted Life Years"[Mesh] OR "prevalence"[tiab] OR "burden"[tiab] OR "cost of illness"[tiab] OR "cost of care"[tiab] OR "mortality"[tiab] OR "disability adjusted life years"[tiab] OR "DALY"[tiab] OR "years of life lost"[tiab] OR "YLL"[tiab] OR "years lived with disability"[tiab] OR "YLD"[tiab])) OR (((((((("Surgical Procedures, Operative"[Mesh] OR "General Surgery"[Mesh] OR "Surgery, Plastic"[Mesh] OR "Plastic Surgery Procedures"[Mesh] OR "surger*"[tiab] OR "plastic surger*"[tiab] OR "reconst* surger*"[tiab] OR "plastic reconstr* surger*"[tiab] OR "surgi* inter*"[tiab]) AND ("Wounds and Injuries"[Mesh] OR "War-Related Injuries"[Mesh] OR "injury"[tiab] OR "traumatic injury"[tiab] OR "post traumatic injury"[tiab] OR ((("Wounds and Injuries"[Mesh] AND "Natural Disasters"[Mesh]) OR ("Wounds and Injuries"[Mesh] AND "War-Related Injuries"[Mesh])) OR ("Wounds and Injuries"[Mesh] AND "Accidents"[Mesh])) OR ((("Wounds and Injuries"[Mesh] AND "Accidents, Traffic"[Mesh]) OR ("Wounds and Injuries"[Mesh] AND "Accidents, Occupational"[Mesh])) OR ("Wounds and Injuries"[Mesh] AND "Accidents, Home"[Mesh])) OR "injur*"[tiab] OR "traum* injur*"[tiab] OR "post traum* injur*"[tiab] OR "postwar injur*"[tiab] OR "traffic accident* injur*"[tiab] OR "home accident*"[tiab] OR "occupation* accident*"[tiab])) OR (("Surgical Procedures, Operative"[Mesh] OR "General Surgery"[Mesh] OR "Surgery, Plastic"[Mesh] OR "Plastic Surgery Procedures"[Mesh] OR "surger*"[tiab] OR "plastic surger*"[tiab] OR "reconst* surger*"[tiab] OR "plastic reconstr* surger*"[tiab] OR "surgi* inter*"[tiab]) AND ("Burns"[MeSH] OR "burn*"[tiab] OR "burn* injur*"[tiab] OR "burn* traum*"[tiab]))) OR (("Surgical Procedures, Operative"[Mesh] OR "General Surgery"[Mesh] OR "Surgery, Plastic"[Mesh] OR "Plastic Surgery Procedures"[Mesh] OR "surger*"[tiab] OR "plastic surger*"[tiab] OR "reconst* surger*"[tiab] OR "plastic reconstr* surger*"[tiab] OR "surgi* inter*"[tiab]) AND ("Congenital Abnormalities"[Mesh]))) OR (("Surgical Procedures, Operative"[Mesh] OR "General Surgery"[Mesh] OR "Surgery, Plastic"[Mesh] OR "Plastic Surgery Procedures"[Mesh] OR "surger*"[tiab] OR "plastic surger*"[tiab] OR "reconst* surger*"[tiab] OR "plastic reconstr* surger*"[tiab] OR "surgi* inter*"[tiab]) AND ("mutila*"[tiab] OR "mutila* injur*"[tiab]))) OR (("Surgical Procedures, Operative"[Mesh] OR "General Surgery"[Mesh] OR "Surgery, Plastic"[Mesh] OR "Plastic Surgery Procedures"[Mesh] OR "surger*"[tiab] OR "plastic surger*"[tiab] OR "reconst* surger*"[tiab] OR "plastic reconstr* surger*"[tiab] OR "surgi* inter*"[tiab]) AND ("disab*"[tiab] OR "disab* injur*"[tiab] OR "disab* patho*"[tiab]))) OR (("Surgical Procedures, Operative"[Mesh] OR "General Surgery"[Mesh] OR "Surgery, Plastic"[Mesh] OR "Plastic Surgery Procedures"[Mesh] OR "surger*"[tiab] OR "plastic surger*"[tiab] OR "reconst* surger*"[tiab] OR "plastic reconstr* surger*"[tiab] OR "surgi* inter*"[tiab]) AND ("deform*"[tiab] OR "deform* injur*"[tiab] OR "deform* patho*"[tiab]))) AND ("Health Services Accessibility"[Mesh] OR "health* serv* access*"[tiab] OR "Healthcare Disparities"[Mesh] OR "health* dispar*"[tiab] OR "Health Inequities"[Mesh] OR "health* ineq*"[tiab] OR "Socioeconomic Disparities in Health"[Mesh] OR "Health Disparate Minority and Vulnerable Populations"[Mesh] OR "barrier* to care*"[tiab]))) AND ("Africa South of the Sahara"[Mesh] OR "Sub-Saharan Africa"[tiab] OR "Sub Saharan Africa"[tiab] OR "Sub-Sahara Africa"[tiab] OR "Sub Sahara Africa"[tiab] OR "sub-Saharan Africa"[tiab] OR "sub Saharan Africa"[tiab] OR "ssa"[tiab] OR "SSA"[tiab] OR "Afric*"[tiab] OR "Angola"[Mesh] OR "Angola"[tiab] OR "Benin"[Mesh] OR "Benin"[tiab] OR "Dahomey"[tiab] OR "Botswana"[Mesh] OR "Botswana"[tiab] OR "Bechuanaland"[tiab] OR "Burkina Faso"[Mesh] OR "Burkina Faso"[tiab] OR "Upper Volta"[tiab] OR "Burundi"[Mesh] OR "Burundi"[tiab] OR "Urundi"[tiab] OR "Cabo Verde"[Mesh] OR "Cabo Verde"[tiab] OR "Cape Verde"[tiab] OR "Cameroon"[Mesh] OR "Cameroon"[tiab] OR "Central African Republic"[Mesh] OR "Central African Republic"[tiab] OR "Ubangi-Shari"[tiab] OR "Chad"[Mesh] OR "Chad"[tiab] OR "Comoros"[Mesh] OR "Comoros"[tiab] OR "Congo"[Mesh] OR "Congo"[tiab] OR "Republic of the Congo"[tiab] OR "Congo-Brazzaville"[tiab] OR "Cote d'Ivoire"[Mesh] OR "Cote d'Ivoire"[tiab] OR "Ivory Coast"[tiab] OR "Democratic Republic of the Congo"[Mesh] OR "Democratic Republic of the Congo"[tiab] OR "DRC"[tiab] OR "Zaire"[tiab] OR "Belgian Congo"[tiab] OR "Djibouti"[Mesh] OR "Djibouti"[tiab] OR "French Somaliland"[tiab] OR "Equatorial Guinea"[Mesh] OR "Equatorial Guinea"[tiab] OR "Spanish Guinea"[tiab] OR "Eritrea"[Mesh] OR "Eritrea"[tiab] OR "Italian Eritrea"[tiab:~0] OR "Eswatini"[Mesh] OR "Eswatini"[tiab] OR "Swaziland"[tiab] OR "Ethiopia"[Mesh] OR "Ethiopia"[tiab] OR "Abyssinia"[tiab] OR "Gabon"[Mesh] OR "Gabon"[tiab] OR "Gambia"[Mesh] OR "Gambia"[tiab] OR "Ghana"[Mesh] OR "Ghana"[tiab] OR "Gold Coast"[tiab] OR "Guinea"[Mesh] OR "Guinea"[tiab] OR "French Guinea"[tiab] OR "Guinea-Bissau"[Mesh] OR "Guinea-Bissau"[tiab] OR "Portuguese Guinea"[tiab] OR "Kenya"[Mesh] OR "Kenya"[tiab] OR "British East Africa"[tiab] OR "Lesotho"[Mesh] OR "Lesotho"[tiab] OR "Basutoland"[tiab] OR "Liberia"[Mesh] OR "Liberia"[tiab] OR "Madagascar"[Mesh] OR "Madagascar"[tiab] OR "Malawi"[Mesh] OR "Malawi"[tiab] OR "Nyasaland"[tiab] OR "Mali"[Mesh] OR "Mali"[tiab] OR "French Sudan"[tiab] OR "Mauritania"[Mesh] OR "Mauritania"[tiab] OR "Mauritius"[Mesh] OR "Mauritius"[tiab] OR "Mozambique"[Mesh] OR "Mozambique"[tiab] OR "Portuguese East Africa"[tiab] OR "Namibia"[Mesh] OR "Namibia"[tiab] OR "South West Africa"[tiab] OR "Niger"[Mesh] OR "Niger"[tiab] OR "Nigeria"[Mesh] OR "Nigeria"[tiab] OR "Rwanda"[Mesh] OR "Rwanda"[tiab] OR "Ruanda"[tiab] OR "Sao Tome and Principe"[Mesh] OR "Sao Tome and Principe"[tiab] OR "Senegal"[Mesh] OR "Senegal"[tiab] OR "Seychelles"[Mesh] OR "Seychelles"[tiab] OR "Sierra Leone"[Mesh] OR "Sierra Leone"[tiab] OR "Somalia"[Mesh] OR "Somalia"[tiab] OR "Somali Republic"[tiab] OR "Italian Somaliland"[tiab:~0] OR "British Somaliland"[tiab] OR "South Sudan"[Mesh] OR "South Sudan"[tiab] OR "Sudan"[Mesh] OR "Sudan"[tiab] OR "Anglo-Egyptian Sudan"[tiab] OR "Tanzania"[Mesh] OR "Tanzania"[tiab] OR "Tanganyika"[tiab] OR "Zanzibar"[tiab] OR "Togo"[Mesh] OR "Togo"[tiab] OR "Togoland"[tiab] OR "Uganda"[Mesh] OR "Uganda"[tiab] OR "Zambia"[Mesh] OR "Zambia"[tiab] OR "Northern Rhodesia"[tiab] OR "Zimbabwe"[Mesh] OR "Zimbabwe"[tiab] OR "Rhodesia"[tiab] OR "Southern Rhodesia"[tiab])

## **Embase**

*(*Result numbers based on final combination search conducted on 21 October 2024)*

| Search # | Query | Results |
| --- | --- | --- |
| 1 | 'surgery'/exp OR 'surger*':ti,ab OR 'reconstructive surgery'/exp OR 'reconst* surger*':ti,ab OR 'plastic surgery'/exp OR 'plastic surger*':ti,ab OR 'plastic reconst* surger*':ti,ab | 6,955,134 |
| 2 | 'injury'/exp OR 'injur*':ti,ab OR 'wound'/exp OR 'wound':ti,ab OR 'traum* injur*':ti,ab OR 'post traum* injur*':ti,ab | 3,533,638 |
| 3 | 'burn'/exp OR 'burn*':ti,ab OR 'burn patient'/exp OR 'burn* patient*':ti,ab OR 'burn survivor'/exp OR 'burn* survivor*':ti,ab OR 'burn* injur*':ti,ab OR 'burn* traum*':ti,ab | 201,393 |
| 4 | 'congenital malformation'/exp OR 'congenital mal*':ti,ab OR 'congenital disorder'/exp OR 'congenital dis*':ti,ab OR 'congenital anomal*':ti,ab OR 'congenital ab*':ti,ab OR 'congenital def*':ti,ab OR 'birth def*':ti,ab | 1,970,729 |
| 5 | 'mutila*':ti,ab OR 'mutila* injur*':ti,ab OR 'mutila* patho*':ti,ab | 9,842 |
| 6 | 'disability'/exp OR 'disab*':ti,ab OR 'physical disability'/exp OR 'physical disab*':ti,ab OR 'motor dysfunction'/exp OR 'motor dys*':ti,ab OR 'disab* injur*':ti,ab OR 'disab* patho*':ti,ab | 1,456,244 |
| 7 | 'deformity'/exp OR 'deform*':ti,ab OR 'deform* injur*':ti,ab OR 'deform* patho*':ti,ab | 236,419 |
| 8 | 'prevalence'/exp OR 'prevalence':ti,ab OR 'epidemiology'/exp OR 'epidemiology':ti,ab OR 'cost of illness'/exp OR 'cost of illness':ti,ab OR 'cost of care':ti,ab OR 'mortality'/exp OR 'mortality':ti,ab OR 'disability-adjusted life year'/exp OR 'disability adjusted life year*':ti,ab OR 'daly':ti,ab OR 'year* of life lost':ti,ab OR 'yll':ti,ab OR 'year* lived with disability':ti,ab OR 'yld':ti,ab OR 'disease burden'/exp OR 'burden of disease*':ti,ab OR 'burden':ti,ab | 6,115,898 |
| 9 | 'health care access'/exp OR 'health care access*':ti,ab OR 'health care disparity'/exp OR 'health care dispar*':ti,ab | 153,754 |
| 10 | 'africa south of the sahara'/exp OR 'africa south of the sahara':ti,ab OR 'sub-saharan african'/exp OR 'sub-saharan african':ti,ab OR 'sub saharan africa':ti,ab OR 'sub-saharan africa':ti,ab OR 'ssa':ti,ab OR 'afric*':ti,ab OR 'angola'/exp OR 'angola':ti,ab OR 'benin'/exp OR 'benin':ti,ab OR 'dahomey':ti,ab OR 'botswana'/exp OR 'botswana':ti,ab OR 'botswanan':ti,ab OR 'bechuanaland':ti,ab OR 'burkina faso'/exp OR 'burkina faso':ti,ab OR 'upper volta':ti,ab OR 'burundi'/exp OR 'burundi':ti,ab OR 'urundi':ti,ab OR 'cameroon'/exp OR 'cameroon':ti,ab OR 'cape verde'/exp OR 'cape verde':ti,ab OR 'cabo verde':ti,ab OR 'central african republic'/exp OR 'central african republic':ti,ab OR 'ubangi-shari':ti,ab OR 'chad'/exp OR 'chad':ti,ab OR 'comoros'/exp OR 'comoros':ti,ab OR 'democratic republic of the congo'/exp OR 'democratic republic of the congo':ti,ab OR 'drc':ti,ab OR 'zaire':ti,ab OR 'congo-kinshasa':ti,ab OR 'congo-leopoldville':ti,ab OR 'belgian congo':ti,ab OR 'congo'/exp OR 'congo':ti,ab OR 'republic of the congo':ti,ab OR 'congo-brazzaville':ti,ab OR 'cote d`ivoire'/exp OR 'cote d`ivoire':ti,ab OR 'ivory coast':ti,ab OR 'djibouti'/exp OR 'djibouti':ti,ab OR 'french somaliland':ti,ab OR 'equatorial guinea'/exp OR 'equatorial guinea':ti,ab OR 'spanish guinea':ti,ab OR 'eritrea'/exp OR 'eritrea':ti,ab OR 'italian eritrea':ti,ab OR 'eswatini'/exp OR 'eswatini':ti,ab OR 'swaziland':ti,ab OR 'ethiopia'/exp OR 'ethiopia':ti,ab OR 'abyssinia':ti,ab OR 'gabon'/exp OR 'gabon':ti,ab OR 'gambia'/exp OR 'gambia':ti,ab OR 'ghana'/exp OR 'ghana':ti,ab OR 'gold coast':ti,ab OR 'guinea'/exp OR 'guinea':ti,ab OR 'french guinea':ti,ab OR 'guinea-bissau'/exp OR 'guinea-bissau':ti,ab OR 'portuguese guinea':ti,ab OR 'kenya'/exp OR 'kenya':ti,ab OR 'british east africa':ti,ab OR 'lesotho'/exp OR 'lesotho':ti,ab OR 'basutoland':ti,ab OR 'liberia'/exp OR 'liberia':ti,ab OR 'madagascar'/exp OR 'madagascar':ti,ab OR 'malagasy republic':ti,ab OR 'malawi'/exp OR 'malawi':ti,ab OR 'nyasaland':ti,ab OR 'mali'/exp OR 'mali':ti,ab OR 'french sudan':ti,ab OR 'mauritania'/exp OR 'mauritania':ti,ab OR 'mauritius'/exp OR 'mauritius':ti,ab OR 'mozambique'/exp OR 'mozambique':ti,ab OR 'portuguese east africa':ti,ab OR 'namibia'/exp OR 'namibia':ti,ab OR 'south west africa':ti,ab OR 'german south west africa':ti,ab OR 'niger'/exp OR 'niger':ti,ab OR 'nigeria'/exp OR 'nigeria':ti,ab OR 'rwanda'/exp OR 'rwanda':ti,ab OR 'ruanda-urundi':ti,ab OR 'ruanda':ti,ab OR 'sao tome and principe'/exp OR 'sao tome and principe':ti,ab OR 'senegal'/exp OR 'senegal':ti,ab OR 'seychelles'/exp OR 'seychelles':ti,ab OR 'sierra leone'/exp OR 'sierra leone':ti,ab OR 'somalia'/exp OR 'somalia':ti,ab OR 'somali republic':ti,ab OR 'somaliland':ti,ab OR 'british somaliland':ti,ab OR 'italian somaliland':ti,ab OR 'south sudan'/exp OR 'south sudan':ti,ab OR 'sudan'/exp OR 'sudan':ti,ab OR 'anglo-egyptian sudan':ti,ab OR 'tanzania'/exp OR 'tanzania':ti,ab OR 'tanganyika':ti,ab OR 'zanzibar':ti,ab OR 'togo'/exp OR 'togo':ti,ab OR 'togoland':ti,ab OR 'uganda'/exp OR 'uganda':ti,ab OR 'zambia'/exp OR 'zambia':ti,ab OR 'northern rhodesia':ti,ab OR 'zimbabwe'/exp OR 'zimbabwe':ti,ab OR 'rhodesia':ti,ab OR 'southern rhodesia':ti,ab | 820,720 |
| 11 | 1 AND 2 | 1,394,062 |
| 12 | 1 AND 3 | 50,228 |
| 13 | 1 AND 4 | 600,599 |
| 14 | 1 AND 5 | 4,933 |
| 15 | 1 AND 6 | 325,386 |
| 16 | 1 AND 7 | 94,395 |
| 17 | 11 OR 12 OR 13 OR 14 OR 15 OR 16 | 2,103,356 |
| 18 | 17 AND 8 | 418,291 |
| 19 | 17 AND 9 | 4,155 |
| 20 | 18 OR 19 | 420,405 |
| 21 | 20 AND 10 | 7,848 |

**Final Embase Query:**

('africa south of the sahara'/exp OR 'africa south of the sahara':ti,ab OR 'sub-saharan african'/exp OR 'sub-saharan african':ti,ab OR 'sub saharan africa':ti,ab OR 'sub-saharan africa':ti,ab OR 'ssa':ti,ab OR 'afric*':ti,ab OR 'angola'/exp OR 'angola':ti,ab OR 'benin'/exp OR 'benin':ti,ab OR 'dahomey':ti,ab OR 'botswana'/exp OR 'botswana':ti,ab OR 'botswanan':ti,ab OR 'bechuanaland':ti,ab OR 'burkina faso'/exp OR 'burkina faso':ti,ab OR 'upper volta':ti,ab OR 'burundi'/exp OR 'burundi':ti,ab OR 'urundi':ti,ab OR 'cameroon'/exp OR 'cameroon':ti,ab OR 'cape verde'/exp OR 'cape verde':ti,ab OR 'cabo verde':ti,ab OR 'central african republic'/exp OR 'central african republic':ti,ab OR 'ubangi-shari':ti,ab OR 'chad'/exp OR 'chad':ti,ab OR 'comoros'/exp OR 'comoros':ti,ab OR 'democratic republic of the congo'/exp OR 'democratic republic of the congo':ti,ab OR 'drc':ti,ab OR 'zaire':ti,ab OR 'congo-kinshasa':ti,ab OR 'congo-leopoldville':ti,ab OR 'belgian congo':ti,ab OR 'congo'/exp OR 'congo':ti,ab OR 'republic of the congo':ti,ab OR 'congo-brazzaville':ti,ab OR 'cote d`ivoire'/exp OR 'cote d`ivoire':ti,ab OR 'ivory coast':ti,ab OR 'djibouti'/exp OR 'djibouti':ti,ab OR 'french somaliland':ti,ab OR 'equatorial guinea'/exp OR 'equatorial guinea':ti,ab OR 'spanish guinea':ti,ab OR 'eritrea'/exp OR 'eritrea':ti,ab OR 'italian eritrea':ti,ab OR 'eswatini'/exp OR 'eswatini':ti,ab OR 'swaziland':ti,ab OR 'ethiopia'/exp OR 'ethiopia':ti,ab OR 'abyssinia':ti,ab OR 'gabon'/exp OR 'gabon':ti,ab OR 'gambia'/exp OR 'gambia':ti,ab OR 'ghana'/exp OR 'ghana':ti,ab OR 'gold coast':ti,ab OR 'guinea'/exp OR 'guinea':ti,ab OR 'french guinea':ti,ab OR 'guinea-bissau'/exp OR 'guinea-bissau':ti,ab OR 'portuguese guinea':ti,ab OR 'kenya'/exp OR 'kenya':ti,ab OR 'british east africa':ti,ab OR 'lesotho'/exp OR 'lesotho':ti,ab OR 'basutoland':ti,ab OR 'liberia'/exp OR 'liberia':ti,ab OR 'madagascar'/exp OR 'madagascar':ti,ab OR 'malagasy republic':ti,ab OR 'malawi'/exp OR 'malawi':ti,ab OR 'nyasaland':ti,ab OR 'mali'/exp OR 'mali':ti,ab OR 'french sudan':ti,ab OR 'mauritania'/exp OR 'mauritania':ti,ab OR 'mauritius'/exp OR 'mauritius':ti,ab OR 'mozambique'/exp OR 'mozambique':ti,ab OR 'portuguese east africa':ti,ab OR 'namibia'/exp OR 'namibia':ti,ab OR 'south west africa':ti,ab OR 'german south west africa':ti,ab OR 'niger'/exp OR 'niger':ti,ab OR 'nigeria'/exp OR 'nigeria':ti,ab OR 'rwanda'/exp OR 'rwanda':ti,ab OR 'ruanda-urundi':ti,ab OR 'ruanda':ti,ab OR 'sao tome and principe'/exp OR 'sao tome and principe':ti,ab OR 'senegal'/exp OR 'senegal':ti,ab OR 'seychelles'/exp OR 'seychelles':ti,ab OR 'sierra leone'/exp OR 'sierra leone':ti,ab OR 'somalia'/exp OR 'somalia':ti,ab OR 'somali republic':ti,ab OR 'somaliland':ti,ab OR 'british somaliland':ti,ab OR 'italian somaliland':ti,ab OR 'south sudan'/exp OR 'south sudan':ti,ab OR 'sudan'/exp OR 'sudan':ti,ab OR 'anglo-egyptian sudan':ti,ab OR 'tanzania'/exp OR 'tanzania':ti,ab OR 'tanganyika':ti,ab OR 'zanzibar':ti,ab OR 'togo'/exp OR 'togo':ti,ab OR 'togoland':ti,ab OR 'uganda'/exp OR 'uganda':ti,ab OR 'zambia'/exp OR 'zambia':ti,ab OR 'northern rhodesia':ti,ab OR 'zimbabwe'/exp OR 'zimbabwe':ti,ab OR 'rhodesia':ti,ab OR 'southern rhodesia':ti,ab) AND ((('prevalence'/exp OR 'prevalence':ti,ab OR 'epidemiology'/exp OR 'epidemiology':ti,ab OR 'cost of illness'/exp OR 'cost of illness':ti,ab OR 'cost of care':ti,ab OR 'mortality'/exp OR 'mortality':ti,ab OR 'disability-adjusted life year'/exp OR 'disability adjusted life year*':ti,ab OR 'daly':ti,ab OR 'year* of life lost':ti,ab OR 'yll':ti,ab OR 'year* lived with disability':ti,ab OR 'yld':ti,ab OR 'disease burden'/exp OR 'burden of disease*':ti,ab OR 'burden':ti,ab) AND ((('surgery'/exp OR 'surger*':ti,ab OR 'reconstructive surgery'/exp OR 'reconst* surger*':ti,ab OR 'plastic surgery'/exp OR 'plastic surger*':ti,ab OR 'plastic reconst* surger*':ti,ab) AND ('injury'/exp OR 'injur*':ti,ab OR 'wound'/exp OR 'wound':ti,ab OR 'traum* injur*':ti,ab OR 'post traum* injur*':ti,ab)) OR (('surgery'/exp OR 'surger*':ti,ab OR 'reconstructive surgery'/exp OR 'reconst* surger*':ti,ab OR 'plastic surgery'/exp OR 'plastic surger*':ti,ab OR 'plastic reconst* surger*':ti,ab) AND ('burn'/exp OR 'burn*':ti,ab OR 'burn patient'/exp OR 'burn* patient*':ti,ab OR 'burn survivor'/exp OR 'burn* survivor*':ti,ab OR 'burn* injur*':ti,ab OR 'burn* traum*':ti,ab)) OR (('surgery'/exp OR 'surger*':ti,ab OR 'reconstructive surgery'/exp OR 'reconst* surger*':ti,ab OR 'plastic surgery'/exp OR 'plastic surger*':ti,ab OR 'plastic reconst* surger*':ti,ab) AND ('congenital malformation'/exp OR 'congenital mal*':ti,ab OR 'congenital disorder'/exp OR 'congenital dis*':ti,ab OR 'congenital anomal*':ti,ab OR 'congenital ab*':ti,ab OR 'congenital def*':ti,ab OR 'birth def*':ti,ab)) OR (('surgery'/exp OR 'surger*':ti,ab OR 'reconstructive surgery'/exp OR 'reconst* surger*':ti,ab OR 'plastic surgery'/exp OR 'plastic surger*':ti,ab OR 'plastic reconst* surger*':ti,ab) AND ('mutila*':ti,ab OR 'mutila* injur*':ti,ab OR 'mutila* patho*':ti,ab)) OR (('surgery'/exp OR 'surger*':ti,ab OR 'reconstructive surgery'/exp OR 'reconst* surger*':ti,ab OR 'plastic surgery'/exp OR 'plastic surger*':ti,ab OR 'plastic reconst* surger*':ti,ab) AND ('disability'/exp OR 'disab*':ti,ab OR 'physical disability'/exp OR 'physical disab*':ti,ab OR 'motor dysfunction'/exp OR 'motor dys*':ti,ab OR 'disab* injur*':ti,ab OR 'disab* patho*':ti,ab)) OR (('surgery'/exp OR 'surger*':ti,ab OR 'reconstructive surgery'/exp OR 'reconst* surger*':ti,ab OR 'plastic surgery'/exp OR 'plastic surger*':ti,ab OR 'plastic reconst* surger*':ti,ab) AND ('deformity'/exp OR 'deform*':ti,ab OR 'deform* injur*':ti,ab OR 'deform* patho*':ti,ab)))) OR (('health care access'/exp OR 'health care access*':ti,ab OR 'health care disparity'/exp OR 'health care dispar*':ti,ab) AND ((('surgery'/exp OR 'surger*':ti,ab OR 'reconstructive surgery'/exp OR 'reconst* surger*':ti,ab OR 'plastic surgery'/exp OR 'plastic surger*':ti,ab OR 'plastic reconst* surger*':ti,ab) AND ('injury'/exp OR 'injur*':ti,ab OR 'wound'/exp OR 'wound':ti,ab OR 'traum* injur*':ti,ab OR 'post traum* injur*':ti,ab)) OR (('surgery'/exp OR 'surger*':ti,ab OR 'reconstructive surgery'/exp OR 'reconst* surger*':ti,ab OR 'plastic surgery'/exp OR 'plastic surger*':ti,ab OR 'plastic reconst* surger*':ti,ab) AND ('burn'/exp OR 'burn*':ti,ab OR 'burn patient'/exp OR 'burn* patient*':ti,ab OR 'burn survivor'/exp OR 'burn* survivor*':ti,ab OR 'burn* injur*':ti,ab OR 'burn* traum*':ti,ab)) OR (('surgery'/exp OR 'surger*':ti,ab OR 'reconstructive surgery'/exp OR 'reconst* surger*':ti,ab OR 'plastic surgery'/exp OR 'plastic surger*':ti,ab OR 'plastic reconst* surger*':ti,ab) AND ('congenital malformation'/exp OR 'congenital mal*':ti,ab OR 'congenital disorder'/exp OR 'congenital dis*':ti,ab OR 'congenital anomal*':ti,ab OR 'congenital ab*':ti,ab OR 'congenital def*':ti,ab OR 'birth def*':ti,ab)) OR (('surgery'/exp OR 'surger*':ti,ab OR 'reconstructive surgery'/exp OR 'reconst* surger*':ti,ab OR 'plastic surgery'/exp OR 'plastic surger*':ti,ab OR 'plastic reconst* surger*':ti,ab) AND ('mutila*':ti,ab OR 'mutila* injur*':ti,ab OR 'mutila* patho*':ti,ab)) OR (('surgery'/exp OR 'surger*':ti,ab OR 'reconstructive surgery'/exp OR 'reconst* surger*':ti,ab OR 'plastic surgery'/exp OR 'plastic surger*':ti,ab OR 'plastic reconst* surger*':ti,ab) AND ('disability'/exp OR 'disab*':ti,ab OR 'physical disability'/exp OR 'physical disab*':ti,ab OR 'motor dysfunction'/exp OR 'motor dys*':ti,ab OR 'disab* injur*':ti,ab OR 'disab* patho*':ti,ab)) OR (('surgery'/exp OR 'surger*':ti,ab OR 'reconstructive surgery'/exp OR 'reconst* surger*':ti,ab OR 'plastic surgery'/exp OR 'plastic surger*':ti,ab OR 'plastic reconst* surger*':ti,ab) AND ('deformity'/exp OR 'deform*':ti,ab OR 'deform* injur*':ti,ab OR 'deform* patho*':ti,ab)))))

## **Web of Science (Core Collection)**

### *(*Result numbers based on final combination search conducted on 21 October 2024)*

| Search # | Query | Results |
| --- | --- | --- |
| 1 | TS=(surger* OR "surgical procedure*" OR "operat* procedure*" OR "general surger*" OR "plastic surger*" OR "plastic surger* procedure*" OR "reconst* surgery" OR "plastic reconstr* surger*" OR "plastic reconstr* procedure*" OR "surgical inter*" ) | 1,683,184 |
| 2 | TS=(wound* OR injur* OR "traum* injur*" OR "post traum* injur*" OR "war injur*" OR "post war injur*") | 1,524,932 |
| 3 | TS=(burn* OR "burn injur*" OR "burn* traum*") | 286,796 |
| 4 | (((((TS=("congenital mal*" OR "congenital abnor*" OR "congenital anomal*" OR "congenital def*" OR "birth def*")) OR TS=(congenital NEAR/15 mal*)) OR TS=(congenital NEAR/15 abnor*)) OR TS=(congenital NEAR/15 anomal*)) OR TS=(congenital NEAR/15 def*)) OR TS=(birth NEAR/15 def*) | 139,930 |
| 5 | TS=(mutila* OR "mutila* injur*" OR "mutila* patho*" OR "mutila* traum*") | 9,366 |
| 6 | TS=(disab* OR "disab* injur*" OR "disab* patho*" OR "disab* traum*") | 413,332 |
| 7 | TS=(deform* OR "deform* injur*" OR "deform* patho*" OR "deform* traum*") | 797,382 |
| 8 | (((TS=(prevalence OR epidemiology OR "cost of illness*" OR mortality OR "disability adjusted life years" OR burden OR "cost of care" OR "DALY" OR "years of life lost" OR "YLL" OR "years lived with disability" OR "YLD")) OR TS=(cost NEAR/15 illness*)) OR TS=(cost NEAR/15 care)) OR TS=(disability NEAR/15 "life years") OR TS=(years NEAR/15 "life lost") OR TS=(years NEAR/15 "lived with disability") | 3,279,249 |
| 9 | TS=("health* serv* access*" OR "health care access*" OR "health* dispar*" OR "health care dispar*" OR "health* ineq*" OR "socioeconomic disparit* in health" OR "health disparate minority and vulnerable population*" OR "barrier* to care*") | 63,489 |
| 10 | TS=(("Africa South of the Sahara" OR "Sub-Saharan Africa" OR "Sub Saharan Africa" OR "Sub-Sahara Africa" OR "Sub Sahara Africa" OR "sub-Saharan Africa" OR "sub Saharan Africa" OR "ssa" OR "SSA" OR "Afric*") OR ("Angola") OR ("Benin" OR "Dahomey") OR ("Botswana" OR "Botswan*" OR "Bechuanaland") OR ("Burkina Faso" OR "Upper Volta") OR ("Burundi" OR "Urundi") OR ("Cabo Verde" OR "Cape Verde") OR ("Cameroon") OR ("Central African Republic" OR "Ubangi-Shari") OR ("Chad") OR ("Comoros") OR ("Congo" OR "Republic of the Congo" OR "Congo-Brazzaville") OR ("Cote d'Ivoire" OR "Ivory Coast") OR ("Democratic Republic of the Congo" OR "DRC" OR "Zaire" OR "Belgian Congo" OR "Congo-Kinshasa" OR "Congo-Leopoldville") OR ("Djibouti" OR "French Somaliland") OR ("Equatorial Guinea" OR "Spanish Guinea") OR ("Eritrea" OR "Italian Eritrea") OR ("Eswatini" OR "Swaziland") OR ("Ethiopia" OR "Abyssinia") OR ("Gabon") OR ("Gambia") OR ("Ghana" OR "Gold Coast") OR ("Guinea" OR "French Guinea") OR ("Guinea-Bissau" OR "Portuguese Guinea") OR ("Kenya" OR "British East Africa") OR ("Lesotho" OR "Basutoland") OR ("Liberia") OR ("Madagascar") OR ("Malawi" OR "Nyasaland") OR ("Mali" OR "French Sudan") OR ("Mauritania") OR ("Mauritius") OR ("Mozambique" OR "Portuguese East Africa") OR ("Namibia" OR "South West Africa" OR "German South West Africa") OR ("Niger") OR ("Nigeria") OR ("Rwanda" OR "Ruanda" OR "Ruanda-Urundi") OR ("Sao Tome and Principe") OR ("Senegal") OR ("Seychelles") OR ("Sierra Leone") OR ("Somalia" OR "Somali Republic" OR "Somaliland" OR "British Somaliland" OR "Italian Somaliland") OR ("South Sudan") OR ("Sudan" OR "Anglo-Egyptian Sudan") OR ("Tanzania" OR "Tanganyika" OR "Zanzibar") OR ("Togo" OR "Togoland") OR ("Uganda") OR ("Zambia" OR "Northern Rhodesia") OR ("Zimbabwe" OR "Rhodesia" OR "Southern Rhodesia")) | 1,290,917 |
| 11 | 1 AND 2 | 185,067 |
| 12 | 1 AND 3 | 9,111 |
| 13 | 1 AND 4 | 19,332 |
| 14 | 1 AND 5 | 1,187 |
| 15 | 1 AND 6 | 28,121 |
| 16 | 1 AND 7 | 33,834 |
| 17 | 11 OR 12 OR 13 OR 14 OR 15 OR 16 | 256,546 |
| 18 | 17 AND 8 | 48,456 |
| 19 | 17 AND 9 | 229 |
| 20 | 18 OR 19 | 48,545 |
| 21 | 20 AND 10 | 1,335 |

**Final Web of Science (Core Collection) Query:**

Link for #21: <https://www.webofscience.com/wos/woscc/summary/6700c301-bad2-461a-b503-ab80896f8d6e-0114d9dca9/relevance/1>

TS=(((surger* OR "surgical procedure*" OR "operat* procedure*" OR "general surger*" OR "plastic surger*" OR "plastic surger* procedure*" OR "reconst* surgery" OR "plastic reconstr* surger*" OR "plastic reconstr* procedure*" OR "surgical inter*") AND (wound* OR injur* OR "traum* injur*" OR "post traum* injur*" OR "war injur*" OR "post war injur*" OR burn* OR "burn injur*" OR "burn* traum*" OR "congenital mal*" OR "congenital abnor*" OR "congenital anomal*" OR "congenital def*" OR "birth def*" OR congenital NEAR/15 mal* OR congenital NEAR/15 abnor* OR congenital NEAR/15 anomal* OR congenital NEAR/15 def* OR birth NEAR/15 def* OR mutila* OR "mutila* injur*" OR "mutila* patho*" OR "mutila* traum*" OR disab* OR "disab* injur*" OR "disab* patho*" OR "disab* traum*" OR deform* OR "deform* injur*" OR "deform* patho*" OR "deform* traum*") AND (prevalence OR epidemiology OR "cost of illness*" OR mortality OR "disability adjusted life years" OR burden OR "cost of care" OR "DALY" OR "years of life lost" OR "YLL" OR "years lived with disability" OR "YLD" OR cost NEAR/15 illness* OR cost NEAR/15 care OR disability NEAR/15 "life years" OR years NEAR/15 "life lost" OR years NEAR/15 "lived with disability" OR "health* serv* access*" OR "health care access*" OR "health* dispar*" OR "health care dispar*" OR "health* ineq*" OR "socioeconomic disparit* in health" OR "health disparate minority and vulnerable population*" OR "barrier* to care*")) AND ("Africa South of the Sahara" OR "Sub-Saharan Africa" OR "Sub Saharan Africa" OR "Sub-Sahara Africa" OR "Sub Sahara Africa" OR "sub-Saharan Africa" OR "sub Saharan Africa" OR ssa OR SSA OR Afric* OR "Angola" OR "Benin" OR "Dahomey" OR "Botswana" OR Botswan* OR "Bechuanaland" OR "Burkina Faso" OR "Upper Volta" OR "Burundi" OR "Urundi" OR "Cabo Verde" OR "Cape Verde" OR "Cameroon" OR "Central African Republic" OR "Ubangi-Shari" OR "Chad" OR "Comoros" OR "Congo" OR "Republic of the Congo" OR "Congo-Brazzaville" OR "Cote d'Ivoire" OR "Ivory Coast" OR "Democratic Republic of the Congo" OR DRC OR "Zaire" OR "Belgian Congo" OR "Congo-Kinshasa" OR "Congo-Leopoldville" OR "Djibouti" OR "French Somaliland" OR "Equatorial Guinea" OR "Spanish Guinea" OR "Eritrea" OR "Italian Eritrea" OR "Eswatini" OR "Swaziland" OR "Ethiopia" OR "Abyssinia" OR "Gabon" OR "Gambia" OR "Ghana" OR "Gold Coast" OR "Guinea" OR "French Guinea" OR "Guinea-Bissau" OR "Portuguese Guinea" OR "Kenya" OR "British East Africa" OR "Lesotho" OR "Basutoland" OR "Liberia" OR "Madagascar" OR "Malawi" OR "Nyasaland" OR "Mali" OR "French Sudan" OR "Mauritania" OR "Mauritius" OR "Mozambique" OR "Portuguese East Africa" OR "Namibia" OR "South West Africa" OR "German South West Africa" OR "Niger" OR "Nigeria" OR "Rwanda" OR "Ruanda" OR "Ruanda-Urundi" OR "Sao Tome and Principe" OR "Senegal" OR "Seychelles" OR "Sierra Leone" OR "Somalia" OR "Somali Republic" OR "Somaliland" OR "British Somaliland" OR "Italian Somaliland" OR "South Sudan" OR "Sudan" OR "Anglo-Egyptian Sudan" OR "Tanzania" OR "Tanganyika" OR "Zanzibar" OR "Togo" OR "Togoland" OR "Uganda" OR "Zambia" OR "Northern Rhodesia" OR "Zimbabwe" OR "Rhodesia" OR "Southern Rhodesia"))

## **medRxiv (plus bioRxiv)**

(*Result numbers based on search conducted on 21 October 2024)

| Search # | Query | Results |
| --- | --- | --- |
| 1 | (Surgery OR Plastic Surgery) AND (Injury OR Burns OR Congenital OR Mutilation OR Disability OR Deformity) AND Burden AND Africa | 16 |
| 2 | Surgery AND (Injury OR Burns OR Congenital OR Mutilation OR Disability OR Deformity) AND (Burden OR Africa) | 231 |
| 3 | Surgery AND (Injury OR Burns OR Congenital OR Mutilation OR Disability OR Deformity) AND (Barrier OR Africa) | 249 |
| 4 | Surgery AND (Injury OR Burns OR Congenital OR Mutilation OR Disability OR Deformity) AND (Burden OR Barrier) | 231 |

**Final medRxiv Query:**
Surgery AND (Injury OR Burns OR Congenital OR Mutilation OR Disability OR Deformity) AND (Burden OR Barrier)
